# NKT Cells in Neurological Diseases

**DOI:** 10.3389/fncel.2019.00245

**Published:** 2019-05-29

**Authors:** Yu Cui, Qi Wan

**Affiliations:** Institute of Neuroregeneration and Neurorehabilitation, Qingdao University, Qingdao, China

**Keywords:** nature killer T cells, ischemic stroke, multiple sclerosis, brain tumor, neurodegenerative disease

## Abstract

Natural killer T (NKT) cells are a unique subset of T lymphocytes with the expression of T cell receptor (TCR) and NK cell lineage receptors. These cells can rapidly release large quantities of cytokines and function as a bridge between innate and adaptive immunity. To date, multiple reports have investigated the role of NKT cells under various pathological conditions, such as cancer, autoimmune disease, and infection. Knowledge about NKT cells in neurological diseases is increasing, albeit limited. Here, we review evidence for the involvement of NKT cells in neurological diseases, and discuss immunotherapeutic potential and future study goals. As the development and function of NKT cells become increasingly well understood, the next few years should yield many new insights into NKT cell function, and mechanistic regulation in neurological disorders.

## Introduction

Historically, the brain has long been regarded as an immune-privileged area as a result of the presence of a blood-brain barrier (BBB) and the lack of lymphatic drainage. Researchers focused mainly on neurons or glial cells when investigating the underlying molecular mechanism of neurological disorders (Price et al., 1987; Almad and Maragakis, 2012). However, mounting evidence highlights the importance of neuroinflammation in neurological diseases (Schwartz and Deczkowska, 2016; Fung et al., 2017; Skaper et al., 2018). In addition, the recently discovered glymphatic system and meningeal lymphatics uncovers a way for peripheral immune cells to enter the brain and communicate with resident cells (Da Mesquita et al., 2018; Sun et al., 2018). Thus, the function of peripheral immune cells in neurological diseases should motivate and be investigated by more researchers.

Natural killer T cells are unique CD1d-restricted T lymphocytes that function as a bridge between innate and adaptive immunity. Based on their TCR usage and lipid antigen specificity, NKT cells have been divided into two subpopulations, type I and type II. Both of these subpopulations recognize lipids antigens presented by CD1d (Bendelac et al., 2007; Godfrey et al., 2010; Nishioka et al., 2018). NKT cells account for a small percentage of lymphocytes, but have profound immunomodulatory roles in a variety of diseases, as these cells show both innate, and adaptive immunological features (Taniguchi et al., 2003; Brennan et al., 2013). Given the abundant existence of glycosphingolipids in the brain (Hirabayashi, 2012), we wondered whether and how NKT cells functioned in neurological diseases. In this review, we described NKT cell properties, summarized current reports on the functions of NKT cells in neurological disorders, including ischemic stroke, brain tumor, multiple sclerosis (MS), neurodegenerative disease and other neurological disorders, and discussed immunotherapeutic potential of these cells and the goals of future studies.

## NKT Cell Classification and Effector Function

**FIGURE 1:**
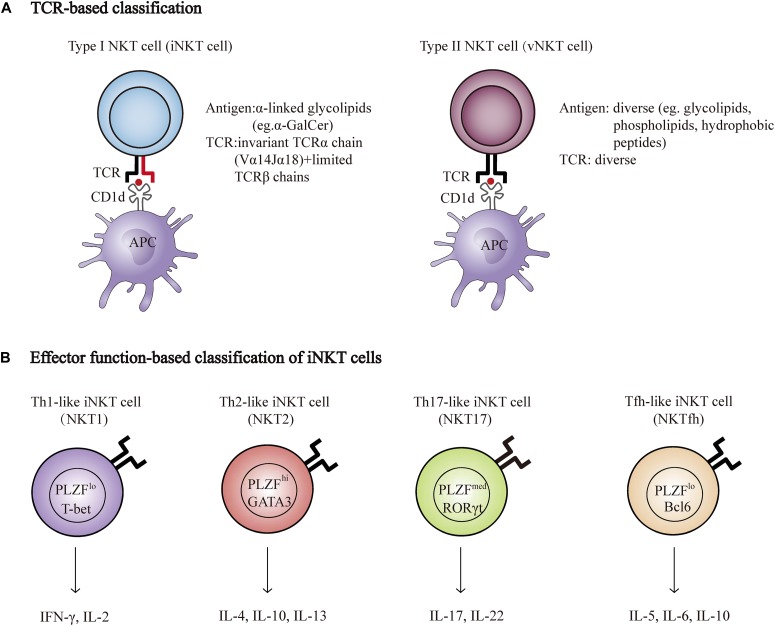
NKT cell classification. **(A)** Properties of Type I (iNKT) and type II NKT cell (vNKT) subpopulations based on the composition of TCR. **(B)** Major subsets of iNKT cells. Transcription factors and cytokines associated with each subset are shown. T-bet, T-box transcription factor; GATA3, GATA-binding protein 3; RORγt, retinoic acid receptor-related orphan receptor-γt; PLZF, promyelocytic leukemia zinc finger protein; Bcl6, B-Cell Lymphoma 6; IFNγ, interferon-γ; IL-2, Interleukin 2.

Natural killer T cells are a specialized subset of T cells that express TCR and NK cell lineage markers, such as NK1.1, NKG2D, and Ly49A. There are two broad categories of NKT cells, type I, and type II. Type I NKT cells, known as invariant NKT cells (iNKT cells) typically express an invariant Vα14-Jα18 TCRα chain and a limited number of non-invariant TCRβ chains that recognize α-galactosylceramide (α-GalCer), a glycosphingolipid isolated from the marine sponge, presented by CD1d. In addition, an increasing number of endogenous antigens, such as isoglobotrihexosylceramide (iGb3) and disialoganglioside (GD3), have also been discovered. Conversely, type II NKT cells use diverse TCRα and β chains that are reactive to more broad antigens, such as glycolipids, phospholipids, and hydrophobic antigens (Bendelac et al., 2007; Godfrey et al., 2010; Nishioka et al., 2018; Figure 1A). Genetic tools have been generated to study NKT cell development and function based on the TCR composition of type I and type II NKT cells. Vα14Jα18 transgenic mice and Jα18^−/−^ mice were used to enrich and delete type I NKT cells, respectively (Bendelac et al., 1996; Cui et al., 1997). In contrast to the Jα18^−/−^ mouse, CD1d^−/−^ mice lack both type I and type II NKT cells, as CD1d is essential for the positive selection of both subsets in the thymus (Chen et al., 1997). Unlike type I NKT cells, whose development and function have been well investigated, the functional role of type II NKT cells is less clear due to the lack of universal and specific staining antibodies, although these cells are more prevalent in humans than type I NKT cells are (Dhodapkar and Kumar, 2017).

Natural killer T cells can quickly secrete large quantities of cytokines, such as interferon-γ (IFN-γ), IL-17, IL-4, and IL-10, which are important for the initiation and regulation of various immune responses (Brennan et al., 2013). Researchers have recently described a new classification system according to the cytokines and transcription factors expressed by type I NKT cells, namely, Th1-like iNKT cell (iNKT1), Th2-like iNKT cell (iNKT2), Th17-like iNKT cell (iNKT17), and Tfh-like iNKT cell (iNKTfh) (Chang et al., 2011; Engel et al., 2016; Figure 1B). The effector function of NKT cells is determined by the differentiation potential of these effector subsets induced via distinct mechanisms of activation. For example, activation with IL-12 and TLR agonists results in the production of Th1-type cytokines instead of Th2-type cytokines (Brigl et al., 2003; Nagarajan and Kronenberg, 2007). In addition, different lipid antigens influence not only the magnitude but also the quality of NKT cell activation (Im et al., 2009).

Natural killer T cells have been reported to participate in a variety of diseases, such as infection, autoimmune disease, and cancer (Dhodapkar and Kumar, 2017). Upon infection, antigenic microbial lipids can be presented by CD1d and then lead to activation-induced cytokine production, which subsequently facilitates the recruitment and activation of other innate, and adaptive immune cells that promote bacterial or viral infection (Brigl et al., 2003). Type I NKT cells have been implicated in antitumor immunity by direct or indirect ways (Godfrey et al., 2018). Notably, chimeric antigen receptor expressing NKT (CAR-NKT) cells and α-Galcer-type I NKT cell-based immunotherapy are being explored (Kriegsmann et al., 2018; Pyaram and Yadav, 2018). During the autoimmune response, certain “self” lipid antigens are presented by antigen presenting cells, which then activate NKT cells to promote inflammatory cytokine production, and aggravate the disease (Wu and Van Kaer, 2009). Despite the classical CD1d-mediated activation of NKT cells in disease progression, non-cognate interactions-related stimuli, including cytokines, damage-associated molecular patterns (DAMPs), and pathogen-associated molecular patterns (PAMPs), also contribute to NKT cell activation, which functions in many diseases (Shimizu et al., 2002; Paget et al., 2007). The detailed molecular mechanism of NKT cell-mediated disease development and progression requires further investigation.

## NKT Cells and Neurological Diseases

### NKT Cells and Ischemic Stroke

Ischemic stroke is one of the leading causes of disability and mortality worldwide, and its incidence is increasing. Early reperfusion remains the effective treatment but can result in secondary damage; thus understanding and alleviating brain damage has become the focus of research (Pan et al., 2007). Typically, most studies have focused on neurons, the cells most sensitive to ischemic-induced injury (Baron et al., 2014). However, mounting evidence has elucidated the critical and diverse roles of immune cells during ischemic stroke (Iadecola and Anrather, 2011; Chamorro et al., 2012), which may offer a novel perspective on immunotherapy for resolving this devastating condition.

Several studies have shown that type I NKT cells accumulate in the ischemic hemisphere in mice or rats by transient occlusion of the middle cerebral artery (tMCAO) (Gelderblom et al., 2009; Lehmann et al., 2014). Similarly, when a cerebral ischemia model was induced by permanent occlusion of the middle cerebral artery (pMCAO), type I NKT cells were observed to infiltrate the blood and brain of induced mice at 24 and 48 h, and this infiltration occurred at an earlier time-point when α-Galcer was administered (Wang et al., 2016). The effects of NKT cells on cerebral stroke severity have also been studied. Wang et al. (2016) discovered that brain water content, cerebral infarct volume, neurological deficit scores and brain edema were significantly increased at 24 h in the pMCAO plus α-Galcer group compared with the pMCAO plus vehicle group. In addition, the production of the proinflammatory cytokines TNFα and IFNγ was increased in the pMCAO plus α-Galcer group. These data suggest that activated type I NKT cells may contribute to brain infarction in cerebral ischemia. In contrast, Kleinschnitz et al. (2010) demonstrated that type I and type II NKT cells are probably of minor importance during the early phase of injury in a model of tMCAO by using CD1d-deficient mice. The reasons for the discrepant effects may result from different NKT cell manipulating methods and mouse models. In the study by Wang et al. (2016) the function of activated type I NKT cells was examined by using a pMCAO mouse model through administration of α-Galcer, whereas in the study by Kleinschnitz et al. (2010) the effects of both type I NKT and type II NKT cell deficiency on brain infarction volume were tested by using a tMCAO model induced in CD1d^−/−^ mice. Further studies discriminating the role of different NKT cell subsets in ischemic stroke are needed.

Infectious complications, particularly pneumonia and urinary tract infections, were reported to be leading causes of death in ischemic stroke patients. The suppression of immune responses after brain ischemia increases the susceptibility to infections, and the underlying molecular mechanisms are largely unknown (Meisel and Meisel, 2011; Chamorro et al., 2012). Emerging evidence indicates that NKT cells function in stroke-induced suppression of the immune response. Wong et al. (2011) discovered that NKT cell-deficient mice (CD1d^−/−^) showed a similar brain infarct size after tMCAO but were more susceptible to poststroke pulmonary infection with greater pulmonary damage, more prominent neutrophil infiltration and decreased survival rate. NKT cells showed restricted crawling and produced more Th2-type cytokines, including IL-10 and IL-5, and less Th1-type cytokines, such as IFN-γ and IL-12 after tMCAO. Selective immunomodulation of type I NKT cells with the specific activator α-Galcer or through the blockade of noradrenergic neurotransmitters protected mice from poststroke infections. The same group conducted another study by delineating the systemic immune profile of stroke patients over time. Consistent with observations in mouse models, a profound activation of type I NKT cells was observed after stroke, which positively correlated with IL-10 production in patients with stroke. The authors proposed that stroke-induced activation of type I NKT cells mediated an immunosuppressive response via the release of IL-10, rendering the patients more susceptible to infections (Wong et al., 2017). These results suggest that the modulation of NKT cell activation, especially enhanced IL-10 production, may prevent poststroke-associated infections, although other explanations of poststroke immunosuppression may exist.

### NKT Cells and Multiple Sclerosis

Multiple sclerosis is a chronic autoimmune disorder of the central nervous system (CNS) in young adults. The pathological hallmarks of MS are extensive demyelination and formation of inflammatory plaques in the spinal cord and brain, which causes irreversible neurological injuries (Frohman et al., 2006). Although the cause of this disease is not entirely clear, autoreactive T cells are considered to be the main attackers on the CNS (Kurschus, 2015). Other immune cells, by affecting myelin-reactive T cell function, may also participate in this pathological condition (Herz et al., 2017).

Numerous studies have highlighted the important role of NKT cells during experimental autoimmune encephalomyelitis (EAE), the most commonly used mouse model of MS. Basically, these studies can be divided into two categories according to the tools used to manipulate NKT cells: genetic tools, such as NKT cell TCR-specific transgenic mice or NKT cell-deficient mice, to enrich or delete NKT cells and NKT cell ligands to trigger the activation of NKT cells. Vα14-Jα281 transgenic mice are protected from EAE in the non-diabetic (NOD) background, and this protection is conferred by an inhibited Th1 response (Mars et al., 2002). However, deletion of NKT cells exhibits conflicting effects on EAE, with some studies showing no effects (Singh et al., 2001; Furlan et al., 2003) and other studies showing disease exacerbation in CD1d-deficient mice (Teige et al., 2004) and in Jα18-deficient mice (Furlan et al., 2003; Oh and Chung, 2011; Denney et al., 2012). These contradictory observations may result from different mouse backgrounds or EAE models (Van Kaer et al., 2015).

**FIGURE 2:**
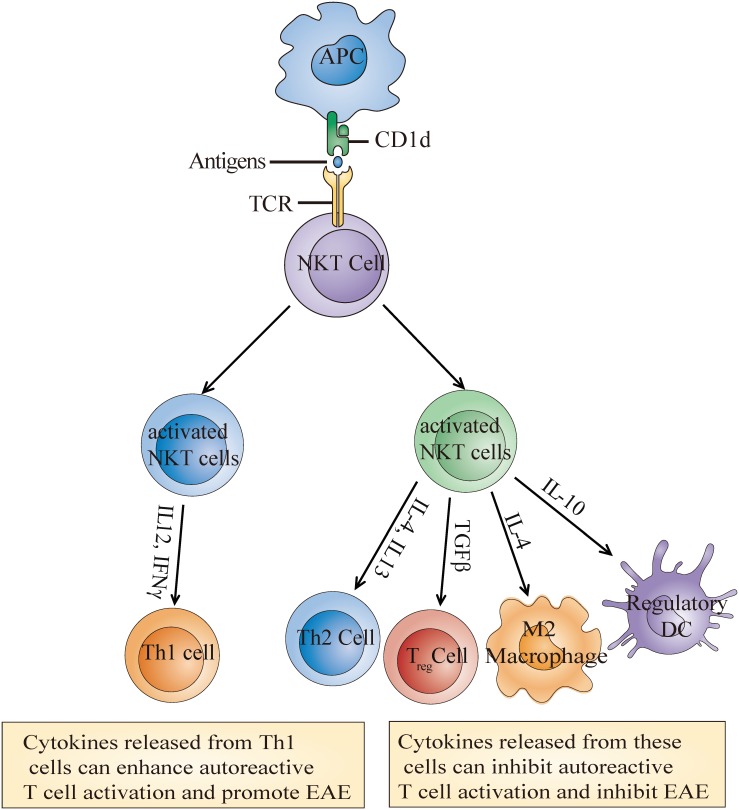
Molecular mechanisms of the NKT cell activation-mediated effects on EAE. Recognition of α-Galcer or its analog presented by CD1d leads to the activation of NKT cells which then secrete multiple cytokines such as IL-4, IL-10, and TGFβ. These cytokines further induce the generation of some immunosuppressive cells or Th1 cells to inhibit or enhance autoreactive T cell activation and eventually alleviate or promote EAE development.

Similar to NKT cell enrichment or deletion, the activation of NKT cells through α-GalCer or its analog also has contrasting effects on EAE outcome. Most studies have shown that the activation of type I NKT cells with α-GalCer or its analog improved EAE outcome (Miyamoto et al., 2001; Singh et al., 2001; Furlan et al., 2003; Kojo et al., 2005; Denney et al., 2012). These improvements are achieved either through indirectly enhancing the Th2 response and/or reducing the Th1 response (Miyamoto et al., 2001; Singh et al., 2001; Furlan et al., 2003) or potentiating the differentiation of immunosuppressive myeloid cell, such as regulatory dendritic cells (DCs) (Kojo et al., 2005), myeloid-derived suppressor cells (MDSCs) (Parekh et al., 2013), and M2 (alternatively activated) macrophages (Denney et al., 2012; Figure 2). Unexpectedly, Qian et al. (2010) demonstrated that high doses of α-GalCer could selectively interacted with CD1d2 expressed on memory T cells instead of activating type I NKT cells, which mainly required CD1d1 expression on APCs, and worsen EAE by directly enhancing Th17 and Th1 differentiation through phosphorylation of STAT3 and activation of NF-κB. Furthermore, another study found that coimmunization of α-GalCer with myelin antigens potentiated EAE in B10. PL mice and prevented EAE in C57BL/6 mice, while prior immunization prevented EAE in both strains. Exacerbation was mediated by promoting Th1 response, and protection was dependent on IL-4 production (Jahng et al., 2001). For type II NKT cells, Jahng et al. discovered that administration of sulfatide, which is only recognized by a subset of type II NKT cells, protected mice from EAE in a CD1d-dependent manner (Jahng et al., 2004). These differences may be due to different mouse backgrounds, as NKT cell populations differ greatly between strains. It is also worth considering that the differences may result from the activation magnitude and treatment time during disease. Regardless of the reason, one conclusion we draw is that the function of NKT cells on EAE mainly depends on the cytokines secreted or factors expressed by NKT cells which can further determine activation or inhibition of peripheral adaptive or innate immune cells, and eventually determine disease outcome.

Several studies have investigated NKT cell number and function in MS patients. Some studies have reported that NKT cell number is decreased in MS patients (Illes et al., 2000; van der Vliet et al., 2001; Demoulins et al., 2003). O’Keeffe et al. (2008) observed an increased frequency of circulating NKT cells in MS patients, while Gausling et al. (2001) did not observe any difference. NKT cells also display distinct cytokine production profiles in MS patients. A study revealed that IL-4 production was increased by CD4^+^ NKT cell clones in RRMS (relapsing-remitting) patients compared to progressive MS patients and control subjects (Araki et al., 2003). Sara et al. found that NKT cells in secondary progressive MS patients displayed pro-inflammatory profiles (De Biasi et al., 2016). These conflicting findings may result from different forms of MS or distinct stages of disease progression. Notably, Stax et al. (2017) reported the surprising finding that sulfatide from myelin or cerebrospinal fluid apoE could directly activate human, but not mouse, type I NKT cells. These findings indicate the important roles of NKT cells in MS, although the detailed molecular mechanism requires further investigation.

Several studies mentioned that the current available drugs targeting MS may exert their actions through targeting NKT cells. It was reported that a great reduction of type I NKT cells in the peripheral blood was associated with the remission state of MS (Araki et al., 2003), and patients who were orally given a low dose of corticosteroid restored the frequency of type I NKT cells (Sakuishi et al., 2010). In addition, type 1 interferon-β (T1IFN-β), the first choice of therapy for MS, have been reported to promote the expansion and function of type I NKT cells to prevent autoimmune disease in pre-clinical models of MS (Gigli et al., 2007). These studies raised the possibility that modulating NKT cell activity might be an alternative way to treat MS.

Altogether, the above findings indicate that NKT cells play diverse roles in MS owing to their cytokine production pattern in certain scenarios. However, some questions remain poorly understood. Instead of investigating how NKT cell activation triggers T cell response, it would be important to determine whether other autoreactive lipids exist in the brain that can activate NKT cells to directly attack the CNS. What are the different roles of type I and type II of NKT cells during MS initiation and progression? Resolving these questions may provide a comprehensive understanding of the effects exerted by NKT cells on MS and expand our strategies for the rational design of NKT-cell-based immunotherapies.

### NKT Cells and Brain Tumors

Brain tumors remain one of the most serious diseases that pose a significant public health threat. Most primary brain tumors are of glial-cell origin, such as glioblastoma, astrocytomas, and oligodendrogliomas (Fu et al., 2014; Gao, 2017). Unlike other kinds of tumors, the treatment of brain tumors is especially difficult due to the blood brain barrier, which restricts drugs from gaining access to the tumors. Although immunotherapies have been considered an effective treatment for tumors, the translation of these strategies to brain tumors is still limited (Brown et al., 2018).

Recent approaches to harness antitumor T cell responses using antibodies of immune checkpoint inhibitors and chimeric antigen receptor (CAR)-T cell immunotherapy have yielded some success (Sanmamed and Chen, 2018). However, due to the complexity of the tumor microenvironment, the application of cancer immunotherapy is limited (Liu et al., 2019). Revealing the functional properties and regulatory mechanisms of immune cells in the tumor microenvironment is critical to overcome the limitations of existing cancer immunotherapy and explore new approaches for cancer immunotherapy. NKT cells, as the bridging system between innate immunity and adaptive immunity, have been implicated in tumor recognition and destruction (Berzins et al., 2011; Godfrey et al., 2018). Type I NKT cells target tumor cells by diverse effective strategies. First, these cells can kill tumor cells by secreting perforin and granzyme B via cytolysis (Nair and Dhodapkar, 2017). Second, activated type I NKT cells secrete various kinds of cytokines to modulate the function of other immune cells such as T cells, B cells and DCs. Third, immunosuppressive cells, including tumor-associated macrophages (TAMs) and MDSCs, could also be targeted by type I NKT cells to indirectly attack tumor cells (McEwen-Smith et al., 2015; Pyaram and Yadav, 2018). Unlike type I NKT cells, type II NKT cells have been reported to inhibit antitumor immunity, although only a few investigations into the role of type II NKT cells in cancer have been published (Godfrey et al., 2018).

Type I NKT cell number and functional properties have been evaluated in glioma patients. Both the number and expanding potential of type I NKT cells in the blood of glioma patients was comparable to those of healthy controls, and these cells could kill glioma in a CD1d-dependent manner, indicating their normal function (Dhodapkar et al., 2004; Teo et al., 2014). Moreover, CD1d expression has been observed in some kinds of brain tumors including medulloblastoma and glioblastoma (Liu et al., 2013; Pyaram and Yadav, 2018). These results suggest that enhancing type I NKT cell function may potentiate antitumor immunity. In support of this notion, studies have observed tumor killing potential by using strategies to induce type I NKT cell activation. Vaccination with irradiated tumor cells pulsed with α-Galcer significantly induced the activation of type I NKT cells and was effective for the treatment of glioma (Hunn et al., 2012). Intracranial administration of NKT cells with the α-Galcer analog 7DW8-5 resulted in an obvious anti-tumor effect (Liu et al., 2013). In addition, co-delivery of tumor-derived exosomes with α-Galcer-loaded DCs improved priming of CD8 T cells and elicited strong anti-tumor immune responses (Liu et al., 2017). To date, the function of type II NKT cells in brain tumors is not understood. These results highlight the necessity of further research to clarify the effects and molecular mechanism of NKT cell subsets in regulating brain tumor immunity, which may offer new therapeutic targets for brain tumor immunotherapy.

### NKT Cells and Neurodegenerative Disease

The increased frequency of neurodegenerative disease can reduce human life expectancy. The most common neurodegenerative diseases are Alzheimer’s disease (AD), Parkinson’s disease (PD), Huntington’s disease (HD), and amyotrophic lateral sclerosis (ALS). Understanding the causes and molecular mechanisms of each disease is desperately required for the effective treatment of neurodegeneration (Heemels, 2016; Gitler et al., 2017).

Despite the well-established role of neuroinflammation in neurodegenerative disease, the function of NKT cells in neurodegeneration is limited (Chen et al., 2016). Notably, Finkelstein et al. reported the role of type I NKT cells in amyotrophic lateral sclerosis (ALS). Type I NKT cells were dramatically accumulated in the spinal cord, spleen and liver of the induced mouse ALS model. Immunomodulation of NKT cells using an analog of α-GalCer delayed motor neuron death and reduced astrogliosis in the spinal cord. Mechanistically, modulation of NKT cells induced a cytokine shift in the liver and facilitated T cell recruitment into the affected spinal cord, leading to a significant prolongation of the life span of mSOD1 mice (Finkelstein et al., 2011). Clearly, more research is required to understand the role of NKT cells in other neurodegenerative diseases considering the high abundance of glycolipids in the brain (Hirabayashi, 2012).

### NKT Cells and CNS Viral Infection

The CNS is classically considered an immune-privileged area. The presence of pathogens can activate tissue resident immune cells via the recognition of PAMPs with pattern recognition receptors (PRRs), resulting in neuroinflammation and inducing subsequent T cell infiltration. Infection of the CNS usually generates efficient immune responses(Ransohoff and Brown, 2012). Mars et al. (2014) demonstrated that type I NKT cells resided in the CNS in the absence of inflammation and their presence dramatically augmented after local viral infection. The infiltrating NKT cells expressed a pro-inflammatory cytokine profile and inhibited the anti-viral responses of CD8 T cells. Accordingly, treatment with exogenous type I NKT cell ligands inhibited the viral-specific CD8 T cell response. These results indicate the involvement of type I NKT cells in viral infection of the CNS.

### NKT Cells and X-Linked Adrenoleukodystrophy (X-ALD)

X-linked adrenoleukodystrophy is a metabolic disorder with the accumulation of very long-chain fatty acids (VLCFA) in plasma and tissues caused by impaired peroxisomal beta-oxidation. The disease is characterized by progressive demyelination within the CNS and adrenal insufficiency (Engelen et al., 2012). In ALD patients, type I NKT cell frequency and CD1d expression on the surface of B cells were slightly decreased (Gautron et al., 2010). Considering the correlation between NKT cell activation ligands and fatty acid metabolism, the function of NKT cells in ALD progression deserves further investigation in mouse models.

### NKT Cells and Myasthenia Gravis

Myasthenia gravis (MG) is an autoimmune disorder of the neuromuscular junction that causes weakness in the skeletal muscles (Thiruppathi et al., 2012). Increased mature NKT cells were observed in the blood of MG patients with thymic hyperplasia, but not in those with thymoma (Suzuki et al., 2005). Immunization with α-GalCer activated type I NKT cells and protected mice from the occurrence of experimental autoimmune myasthenia gravis (EAMG), an animal model of human myasthenia gravis, which may be caused by the increased expansion of Treg cells (Liu et al., 2005; Wang et al., 2015).

### NKT Cells and Neurological Antiphospholipid Syndrome (APS)

Antiphospholipid syndrome is an autoimmune disorder characterized by the presence of antiphospholipid antibodies, such as anticardiolipin antibodies, anti-β2-glycoprotein 1 antibodies, and lupus anticoagulant (Schreiber et al., 2018). APS neurological manifestations have been reported to be associated with some neurological diseases including ischemic stroke and MS (Ricarte et al., 2018; D’Angelo et al., 2019). Of note, cardiolipin is among the reported lipids antigens recognized by CD1d and this recognition may explain the increased number of NKT cells in some autoimmune diseases (Cox et al., 2009; D’Angelo et al., 2019). Wermeling et al. (2010) demonstrated that injection of apoptotic cells decreased the ability of type I NKT cells to produce cytokines, which further increased autoreactive B cells to produce autoreactive antibodies, such as anticardiolipin antibodies and antiphosphorylcholine, although this contrasts with the demonstration that NKT cells could promote B cell responses, and antibody production (Doherty et al., 2018). Considering the close association of antiphospholipid antibodies and some neurological diseases, it will be interesting to modulate NKT cell activity to regulate APS, although a better understanding of NKT cell and APS is needed.

## Conclusion

Recent studies have elucidated novel and striking aspects of NKT cell function and its modulatory roles in disease progression. NKT cell-modulated neurological diseases, particularly ischemic stroke, MS and glioblastoma have been described (Table 1). Despite major advances in the understanding of NKT cell effector function in neurological disorders, there is still much to learn about. First, as more is learned about NKT heterogeneity, different NKT cell subsets should be discriminated in pathological conditions. There is very little knowledge of the role of type II NKT cells in neurological disease which should be the goal of future studies. Second, NKT cell function should be investigated in different disease progression stages. For ischemic stroke, NKT cells do not affect infarct volume but function in poststroke immunosuppression, which is also a very important factor that determines the life span of patients. Third, given the critical role of inflammation in neurodegenerative disease, the function of NKT cells in PD and AD should be explored. Finally, despite the well-established role of cognate recognition-induced activation of NKT cells, the non-cognate interaction-induced activation of NKT cells in disease progression deserves further investigation. If a thorough understanding of the roles and molecular mechanisms of NKT cells in neurological disease is gained, new avenues might be opened in the treatment of neurological disorders.

**Table 1:** Direct evidence of NKT cell functions in neurological diseases.

Neurological diseases (Mouse model)	Tools to study NKT cells	NKT cell subtype	Effects in pathological condition	References
Ischemic stroke				
tMCAO	CD1d^−/−^ mice	Type I and II	Do not affect infarct volume, promote post-stroke immune suppression	Kleinschnitz et al., 2010; Meisel and Meisel, 2011
pMCAO	α-Galcer or its analog injection	Type I	Accelerate infarct volume and brain edema	Wang et al., 2016
tMCAO	α-Galcer injection	Type I	Inhibit post-stroke infection	Meisel and Meisel, 2011
Multiple sclerosis (EAE)	α-Galcer or its analog injection	Type I	Protect mice from EAE by promoting Th2 response and suppress Th1 response	Miyamoto et al., 2001; Singh et al., 2001; Furlan et al., 2003
	α-Galcer injection prior to or at the time of immunization	Type I	Potentiate or prevent EAE depending on mice strain and timing of activation	Jahng et al., 2001
	α-Galcer injection (high dose)	Type I	High dose of α-Galcer potentiate EAE by directly enhancing Th17 response	Qian et al., 2010
	α-Galcer injection at early phase of EAE	Type I	Improve disease outcome by the differentiation of inflammatory monocytes to M2 macrophage	Denney et al., 2012
	α-Galcer injection	Type I	Suppress EAE by inducing a regulatory DC via secretion IL-10	Kojo et al., 2005
	Ja18-KO mice or adoptive NKT cell transfer	Type I	IL-4- and IL-10-producing iNKT cells inhibit the Th1 cell response and protect mice against EAE; No significant effect	Mars et al., 2002; Furlan et al., 2003; Oh and Chung, 2011
	CD1d^−/−^ mice	Type I and II	NKT cells protect mice from EAE by CD1-dependent enhancement of TGFβ production; No significant effect	Singh et al., 2001; Furlan et al., 2003; Teige et al., 2004
	Vα14Jα281 transgenic mice	Type I	Increased NKT cells protect mice from EAE by inhibition of Th1 response	Mars et al., 2002
	Sulfatide injection	Type II	Prevents EAE in a CD1d-dependent manner	Jahng et al., 2004
Brain tumor				
medulloblastoma	Co-injection of NKT with α-Galcer analog	Type I	Intracranial injection of NKT resulted in regression of orthotopic MB xenografts in NOD/SCID mice	Liu et al., 2013
glioma	α-Galcer pulse tumor cells	Type I	α-Galcer pulsed tumor cells stimualtes NKT cells and augment T cell response to kill tumor	Hunn et al., 2012
glioblastoma	α-Galcer pulse dendritic cells	Type I	Co-delivery of tumor-derived exosomes with α-GalCer on a DC-based vaccine showed powerful effects in glioblastoma immunotherapy	Liu et al., 2017
Neurodegenerative disease (ALS-SOD1G93A mice)	α-GalCer analog, PBS57 treatment	Type I	Treatment with PBS57delayed the onset of the disease and prolonged the survival of mSOD1 mice	Finkelstein et al., 2011
CNS viral infection (Theiler’s virus infection)	α-GalCer treatment, Vα14 transgenic mice, Jα18^−/−^ mice	Type I	NKT cells inhibit anti-viral CD8 T cell responsean augmented mortality during acute viral infection	Ransohoff and Brown, 2012
Myasthenia gravis (EAMG)	α-GalCer treatment	Type I	NKT cells protect mice from EAMG by increasing the expansion of Treg cells	Liu et al., 2005; Wang et al., 2015

## Author Contributions

YC was involved in the writing, reading literature, and design of the figures. QW was involved in the overall supervision of the review and editing of the manuscript. All authors gave approval before submission.

## Conflict of Interest Statement

The authors declare that the research was conducted in the absence of any commercial or financial relationships that could be construed as a potential conflict of interest.

## References

[B1] Almad A. A. Maragakis N. J. (2012). Glia: an emerging target for neurological disease therapy. *Stem Cell Res. Ther.* 3:37. 10.1186/scrt128 23021042 PMC3580428

[B2] Araki M. Kondo T. Gumperz J. E. Brenner M. B. Miyake S. Yamamura T. (2003). Th2 bias of CD4+ NKT cells derived from multiple sclerosis in remission. *Int. Immunol.* 15 279–288. 10.1093/intimm/dxg029 12578858

[B3] Baron J. C. Yamauchi H. Fujioka M. Endres M. (2014). Selective neuronal loss in ischemic stroke and cerebrovascular disease. *J. Cereb. Blood Flow Metab.* 34 2–18. 10.1038/jcbfm.2013.188 24192635 PMC3887360

[B4] Bendelac A. Hunziker R. D. Lantz O. (1996). Increased interleukin 4 and immunoglobulin E production in transgenic mice overexpressing NK1 T cells. *J. Exp. Med.* 184 1285–1293. 10.1084/jem.184.4.1285 8879200 PMC2192838

[B5] Bendelac A. Savage P. B. Teyton L. (2007). The biology of NKT cells. *Annu. Rev. Immunol.* 25 297–336. 17150027 10.1146/annurev.immunol.25.022106.141711

[B6] Berzins S. P. Smyth M. J. Baxter A. G. (2011). Presumed guilty: natural killer T cell defects and human disease. *Nat. Rev. Immunol.* 11 131–142. 10.1038/nri2904 21267014

[B7] Brennan P. J. Brigl M. Brenner M. B. (2013). Invariant natural killer T cells: an innate activation scheme linked to diverse effector functions. *Nat. Rev. Immunol.* 13 101–117. 10.1038/nri3369 23334244

[B8] Brigl M. Bry L. Kent S. C. Gumperz J. E. Brenner M. B. (2003). Mechanism of CD1d-restricted natural killer T cell activation during microbial infection. *Nat. Immunol.* 4 1230–1237. 10.1038/ni1002 14578883

[B9] Brown N. F. Carter T. J. Ottaviani D. Mulholland P. (2018). Harnessing the immune system in glioblastoma. *Br. J. Cancer* 119 1171–1181. 10.1038/s41416-018-0258-8 30393372 PMC6251037

[B10] Chamorro A. Meisel A. Planas A. M. Urra X. van de Beek D. Veltkamp R. (2012). The immunology of acute stroke. *Nat. Rev. Neurol.* 8 401–410. 10.1038/nrneurol.2012.98 22664787

[B11] Chang P. P. Barral P. Fitch J. Pratama A. Ma C. S. Kallies A. (2011). Identification of Bcl-6-dependent follicular helper NKT cells that provide cognate help for B cell responses. *Nat. Immunol.* 13 35–43. 10.1038/ni.2166 22120117

[B12] Chen W. W. Zhang X. Huang W. J. (2016). Role of neuroinflammation in neurodegenerative diseases (Review). *Mol. Med. Rep.* 13 3391–3396. 10.3892/mmr.2016.4948 26935478 PMC4805095

[B13] Chen Y. H. Chiu N. M. Mandal M. Wang N. Wang C. R. (1997). Impaired NK1+ T cell development and early IL-4 production in CD1-deficient mice. *Immunity* 6 459–467. 10.1016/s1074-7613(00)80289-7 9133425

[B14] Cox D. Fox L. Tian R. Bardet W. Skaley M. Mojsilovic D. (2009). Determination of cellular lipids bound to human CD1d molecules. *PLoS One* 4:e5325. 10.1371/journal.pone.0005325 19415116 PMC2673035

[B15] Cui J. Shin T. Kawano T. Sato H. Kondo E. Toura I. (1997). Requirement for Valpha14 NKT cells in IL-12-mediated rejection of tumors. *Science* 278 1623–1626. 10.1126/science.278.5343.1623 9374462

[B16] Da Mesquita S. Louveau A. Vaccari A. Smirnov I. Cornelison R. C. Kingsmore K. M. (2018). Functional aspects of meningeal lymphatics in ageing and Alzheimer’s disease. *Nature* 560 185–191. 10.1038/s41586-018-0368-8 30046111 PMC6085146

[B17] D’Angelo C. Franch O. Fernandez-Paredes L. Oreja-Guevara C. Nunez-Beltran M. Comins-Boo A. (2019). Antiphospholipid antibodies overlapping in isolated neurological syndrome and multiple sclerosis: neurobiological insights and diagnostic challenges. *Front. Cell Neurosci.* 13:107. 10.3389/fncel.2019.00107 30941020 PMC6433987

[B18] De Biasi S. Simone A. M. Nasi M. Bianchini E. Ferraro D. Vitetta F. (2016). iNKT cells in secondary progressive multiple sclerosis patients display pro-inflammatory profiles. *Front. Immunol.* 7:555. 27965675 10.3389/fimmu.2016.00555 PMC5127814

[B19] Demoulins T. Gachelin G. Bequet D. Dormont D. (2003). A biased Valpha24+ T-cell repertoire leads to circulating NKT-cell defects in a multiple sclerosis patient at the onset of his disease. *Immunol. Lett.* 90 223–228. 10.1016/j.imlet.2003.09.014 14687729

[B20] Denney L. Kok W. L. Cole S. L. Sanderson S. McMichael A. J. Ho L. P. (2012). Activation of invariant NKT cells in early phase of experimental autoimmune encephalomyelitis results in differentiation of Ly6Chi inflammatory monocyte to M2 macrophages and improved outcome. *J. Immunol.* 189 551–557. 10.4049/jimmunol.1103608 22685310

[B21] Dhodapkar K. M. Cirignano B. Chamian F. Zagzag D. Miller D. C. Finlay J. L. (2004). Invariant natural killer T cells are preserved in patients with glioma and exhibit antitumor lytic activity following dendritic cell-mediated expansion. *Int. J. Cancer* 109 893–899. 10.1002/ijc.20050 15027123

[B22] Dhodapkar M. V. Kumar V. (2017). Type II NKT cells and their emerging role in health and disease. *J. Immunol.* 198 1015–1021. 10.4049/jimmunol.1601399 28115591 PMC5300729

[B23] Doherty D. G. Melo A. M. Moreno-Olivera A. Solomos A. C. (2018). Activation and regulation of B cell responses by invariant natural killer T cells. *Front. Immunol.* 9:1360. 10.3389/fimmu.2018.01360 29967611 PMC6015876

[B24] Engel I. Seumois G. Chavez L. Samaniego-Castruita D. White B. Chawla A. (2016). Innate-like functions of natural killer T cell subsets result from highly divergent gene programs. *Nat. Immunol.* 17 728–739. 10.1038/ni.3437 27089380 PMC4944658

[B25] Engelen M. Kemp S. de Visser M. van Geel B. M. Wanders R. J. Aubourg P. (2012). X-linked adrenoleukodystrophy (X-ALD): clinical presentation and guidelines for diagnosis, follow-up and management. *Orph. J. Rare Dis.* 7:51. 10.1186/1750-1172-7-51 22889154 PMC3503704

[B26] Finkelstein A. Kunis G. Seksenyan A. Ronen A. Berkutzki T. Azoulay D. (2011). Abnormal changes in NKT cells, the IGF-1 axis, and liver pathology in an animal model of ALS. *PLoS One* 6:e22374. 10.1371/journal.pone.0022374 21829620 PMC3149057

[B27] Frohman E. M. Racke M. K. Raine C. S. (2006). Multiple sclerosis–the plaque and its pathogenesis. *N. Engl. J. Med.* 354 942–955. 10.1056/nejmra052130 16510748

[B28] Fu R. Shen Q. Xu P. Luo J. J. Tang Y. (2014). Phagocytosis of microglia in the central nervous system diseases. *Mol. Neurobiol.* 49 1422–1434. 10.1007/s12035-013-8620-6 24395130 PMC4012154

[B29] Fung T. C. Olson C. A. Hsiao E. Y. (2017). Interactions between the microbiota, immune and nervous systems in health and disease. *Nat. Neurosci.* 20 145–155. 10.1038/nn.4476 28092661 PMC6960010

[B30] Furlan R. Bergami A. Cantarella D. Brambilla E. Taniguchi M. Dellabona P. (2003). Activation of invariant NKT cells by alphaGalCer administration protects mice from MOG35-55-induced EAE: critical roles for administration route and IFN-gamma. *Eur. J. Immunol.* 33 1830–1838. 10.1002/eji.200323885 12811843

[B31] Gao H. (2017). Perspectives on dual targeting delivery systems for brain tumors. *J. Neuroimm. Pharmacol.* 12 6–16. 10.1007/s11481-016-9687-4 27270720

[B32] Gausling R. Trollmo C. Hafler D. A. (2001). Decreases in interleukin-4 secretion by invariant CD4(-)CD8(-)V alpha 24J alpha Q T cells in peripheral blood of patientswith relapsing-remitting multiple sclerosis. *Clin. Immunol.* 98 11–17. 10.1006/clim.2000.4942 11141321

[B33] Gautron A. S. Giquel B. Beaudoin L. Autrusseau E. Speak A. Platt F. (2010). Invariant NKT cells in adrenoleukodystrophy patients and mice. *J. Neuroimmunol.* 229 204–211. 10.1016/j.jneuroim.2010.09.003 20920830

[B34] Gelderblom M. Leypoldt F. Steinbach K. Behrens D. Choe C. U. Siler D. A. (2009). Temporal and spatial dynamics of cerebral immune cell accumulation in stroke. *Stroke* 40 1849–1857. 10.1161/STROKEAHA.108.534503 19265055

[B35] Gigli G. Caielli S. Cutuli D. Falcone M. (2007). Innate immunity modulates autoimmunity: type 1 interferon-beta treatment in multiple sclerosis promotes growth and function of regulatory invariant natural killer T cells through dendritic cell maturation. *Immunology* 122 409–417. 10.1111/j.1365-2567.2007.02655.x 17617156 PMC2266024

[B36] Gitler A. D. Dhillon P. Shorter J. (2017). Neurodegenerative disease: models, mechanisms, and a new hope. *Dis. Model Mech.* 10 499–502. 10.1242/dmm.030205 28468935 PMC5451177

[B37] Godfrey D. I. Le Nours J. Andrews D. M. Uldrich A. P. Rossjohn J. (2018). Unconventional T cell targets for cancer immunotherapy. *Immunity* 48 453–473. 10.1016/j.immuni.2018.03.009 29562195

[B38] Godfrey D. I. Stankovic S. Baxter A. G. (2010). Raising the NKT cell family. *Nat. Immunol.* 11 197–206. 10.1038/ni.1841 20139988

[B39] Heemels M. T. (2016). Neurodegenerative diseases. *Nature* 539:179. 10.1038/539179a 27830810

[B40] Herz J. Filiano A. J. Smith A. Yogev N. Kipnis J. (2017). Myeloid cells in the central nervous system. *Immunity* 46 943–956. 10.1016/j.immuni.2017.06.007 28636961 PMC5657250

[B41] Hirabayashi Y. (2012). A world of sphingolipids and glycolipids in the brain–novel functions of simple lipids modified with glucose. *Proc. Jpn. Acad. Ser. B Phys. Biol. Sci.* 88 129–143. 10.2183/pjab.88.129 PMC3406307 22498977

[B42] Hunn M. K. Farrand K. J. Broadley K. W. Weinkove R. Ferguson P. Miller R. J. (2012). Vaccination with irradiated tumor cells pulsed with an adjuvant that stimulates NKT cells is an effective treatment for glioma. *Clin. Cancer Res.* 18 6446–6459. 10.1158/1078-0432.CCR-12-0704 23147997

[B43] Iadecola C. Anrather J. (2011). The immunology of stroke: from mechanisms to translation. *Nat. Med.* 17 796–808. 10.1038/nm.2399 21738161 PMC3137275

[B44] Illes Z. Kondo T. Newcombe J. Oka N. Tabira T. Yamamura T. (2000). Differential expression of NK T cell V alpha 24J alpha Q invariant TCR chain in the lesions of multiple sclerosis and chronic inflammatory demyelinating polyneuropathy. *J. Immunol.* 164 4375–4381. 10.4049/jimmunol.164.8.4375 10754338

[B45] Im J. S. Arora P. Bricard G. Molano A. Venkataswamy M. M. Baine I. (2009). Kinetics and cellular site of glycolipid loading control the outcome of natural killer T cell activation. *Immunity* 30 888–898. 10.1016/j.immuni.2009.03.022 19538930 PMC2719696

[B46] Jahng A. Maricic I. Aguilera C. Cardell S. Halder R. C. Kumar V. (2004). Prevention of autoimmunity by targeting a distinct, noninvariant CD1d-reactive T cell population reactive to sulfatide. *J. Exp. Med.* 199 947–957. 10.1084/jem.20031389 15051763 PMC2211873

[B47] Jahng A. W. Maricic I. Pedersen B. Burdin N. Naidenko O. Kronenberg M. (2001). Activation of natural killer T cells potentiates or prevents experimental autoimmune encephalomyelitis. *J. Exp. Med.* 194 1789–1799. 10.1084/jem.194.12.1789 11748280 PMC2193586

[B48] Kleinschnitz C. Schwab N. Kraft P. Hagedorn I. Dreykluft A. Schwarz T. (2010). Early detrimental T-cell effects in experimental cerebral ischemia are neither related to adaptive immunity nor thrombus formation. *Blood* 115 3835–3842. 10.1182/blood-2009-10-249078 20215643

[B49] Kojo S. Seino K. Harada M. Watarai H. Wakao H. Uchida T. (2005). Induction of regulatory properties in dendritic cells by Valpha14 NKT cells. *J. Immunol.* 175 3648–3655. 10.4049/jimmunol.175.6.3648 16148109

[B50] Kriegsmann K. Kriegsmann M. von Bergwelt-Baildon M. Cremer M. Witzens-Harig M. (2018). NKT cells - new players in CAR cell immunotherapy? *Eur. J. Haematol.* 101 750–757. 10.1111/ejh.13170 30187578

[B51] Kurschus F. C. (2015). T cell mediated pathogenesis in EAE: molecular mechanisms. *Biomed. J.* 38 183–193. 10.4103/2319-4170.155590 25900928

[B52] Lehmann J. Hartig W. Seidel A. Fuldner C. Hobohm C. Grosche J. (2014). Inflammatory cell recruitment after experimental thromboembolic stroke in rats. *Neuroscience* 279 139–154. 10.1016/j.neuroscience.2014.08.023 25168731

[B53] Liu D. Jenkins R. W. Sullivan R. J. (2019). Mechanisms of resistance to immune checkpoint blockade. *Am. J. Clin. Dermatol.* 20 41–54. 10.1007/s40257-018-0389-y 30259383 PMC6358473

[B54] Liu D. F. Song L. P. Brawley V. S. Robison N. Wei J. Gao X. H. (2013). Medulloblastoma expresses CD1d and can be targeted for immunotherapy with NKT cells. *Clin. Immunol.* 149 55–64. 10.1016/j.clim.2013.06.005 23891738 PMC3809126

[B55] Liu H. Chen L. Liu J. Meng H. Zhang R. Ma L. (2017). Co-delivery of tumor-derived exosomes with alpha-galactosylceramide on dendritic cell-based immunotherapy for glioblastoma. *Cancer Lett.* 411 182–190. 10.1016/j.canlet.2017.09.022 28947140

[B56] Liu R. La Cava A. Bai X. F. Jee Y. Price M. Campagnolo D. I. (2005). Cooperation of invariant NKT cells and CD4+CD25+ T regulatory cells in the prevention of autoimmune myasthenia. *J. Immunol.* 175 7898–7904. 10.4049/jimmunol.175.12.7898 16339525

[B57] Mars L. T. Laloux V. Goude K. Desbois S. Saoudi A. Van Kaer L. (2002). Cutting edge: V alpha 14-J alpha 281 NKT cells naturally regulate experimental autoimmune encephalomyelitis in nonobese diabetic mice. *J. Immunol.* 168 6007–6011. 10.4049/jimmunol.168.12.6007 12055208

[B58] Mars L. T. Mas M. Beaudoin L. Bauer J. Leite-de-Moraes M. Lehuen A. (2014). Invariant NKT cells regulate the CD8 T cell response during theiler’s virus infection. *PLoS One* 9:e87717. 10.1371/journal.pone.0087717 24498175 PMC3907484

[B59] McEwen-Smith R. M. Salio M. Cerundolo V. (2015). The regulatory role of invariant NKT cells in tumor immunity. *Cancer Immunol. Res.* 3 425–435. 10.1158/2326-6066.CIR-15-0062 25941354 PMC4430818

[B60] Meisel C. Meisel A. (2011). Suppressing immunosuppression after stroke. *N. Engl. J. Med.* 365 2134–2136. 10.1056/nejmcibr1112454 22129259

[B61] Miyamoto K. Miyake S. Yamamura T. (2001). A. synthetic glycolipid prevents autoimmune encephalomyelitis by inducing TH2 bias of natural killer T cells. *Nature* 413 531–534. 10.1038/35097097 11586362

[B62] Nagarajan N. A. Kronenberg M. (2007). Invariant NKT cells amplify the innate immune response to lipopolysaccharide. *J. Immunol.* 178 2706–2713. 10.4049/jimmunol.178.5.2706 17312112

[B63] Nair S. Dhodapkar M. V. (2017). Natural killer T cells in cancer immunotherapy. *Front. Immunol.* 8:1178. 10.3389/fimmu.2017.01178 29018445 PMC5614937

[B64] Nishioka Y. Masuda S. Tomaru U. Ishizu A. (2018). CD1d-restricted type II NKT cells reactive with endogenous hydrophobic peptides. *Front. Immunol.* 9:548. 10.3389/fimmu.2018.00548 29599785 PMC5862807

[B65] Oh S. J. Chung D. H. (2011). Invariant NKT cells producing IL-4 or IL-10, but not IFN-gamma, inhibit the Th1 response in experimental autoimmune encephalomyelitis, whereas none of these cells inhibits the Th17 response. *J. Immunol.* 186 6815–6821. 10.4049/jimmunol.1003916 21572032

[B66] O’Keeffe J. Gately C. M. Counihan T. Hennessy M. Leahy T. Moran A. P. (2008). T-cells expressing natural killer (NK) receptors are altered in multiple sclerosis and responses to alpha-galactosylceramide are impaired. *J. Neurol. Sci.* 275 22–28. 10.1016/j.jns.2008.07.007 18706662 PMC3116724

[B67] Paget C. Mallevaey T. Speak A. O. Torres D. Fontaine J. Sheehan K. C. (2007). Activation of invariant NKT cells by toll-like receptor 9-stimulated dendritic cells requires type I interferon and charged glycosphingolipids. *Immunity* 27 597–609. 10.1016/j.immuni.2007.08.017 17950005

[B68] Pan J. Konstas A. A. Bateman B. Ortolano G. A. Pile-Spellman J. (2007). Reperfusion injury following cerebral ischemia: pathophysiology, MR imaging, and potential therapies. *Neuroradiology* 49 93–102. 10.1007/s00234-006-0183-z 17177065 PMC1786189

[B69] Parekh V. V. Wu L. Olivares-Villagomez D. Wilson K. T. Van Kaer L. (2013). Activated invariant NKT cells control central nervous system autoimmunity in a mechanism that involves myeloid-derived suppressor cells. *J. Immunol.* 190 1948–1960. 10.4049/jimmunol.1201718 23345328 PMC3577977

[B70] Price D. L. Cork L. C. Struble R. G. Kitt C. A. Walker L. C. Powers R. E. (1987). Dysfunction and death of neurons in human degenerative neurological diseases and in animal models. *Ciba Found. Symp.* 126 30–48. 10.1002/9780470513422.ch3 3556088

[B71] Pyaram K. Yadav V. N. (2018). Advances in NKT cell immunotherapy for glioblastoma. *J. Cancer Sci. Ther.* 10:533. 10.4172/1948-5956.1000533 PMC6108592 30147849

[B72] Qian G. Qin X. Zang Y. Q. Ge B. Guo T. B. Wan B. (2010). High doses of alpha-galactosylceramide potentiate experimental autoimmune encephalomyelitis by directly enhancing Th17 response. *Cell Res.* 20 480–491. 10.1038/cr.2010.6 20084083

[B73] Ransohoff R. M. Brown M. A. (2012). Innate immunity in the central nervous system. *J. Clin. Invest.* 122 1164–1171. 10.1172/jci58644 22466658 PMC3314450

[B74] Ricarte I. F. Dutra L. A. Abrantes F. F. Toso F. F. Barsottini O. G. P. Silva G. S. (2018). Neurologic manifestations of antiphospholipid syndrome. *Lupus* 27 1404–1414. 10.1177/0961203318776110 29768970

[B75] Sakuishi K. Miyake S. Yamamura T. (2010). Role of NK cells and invariant NKT cells in multiple sclerosis. *Results Probl. Cell Differ.* 51 127–147. 10.1007/400_2009_11 19582416

[B76] Sanmamed M. F. Chen L. (2018). A paradigm shift in cancer immunotherapy: from enhancement to normalization. *Cell* 175 313–326. 10.1016/j.cell.2018.09.035 30290139 PMC6538253

[B77] Schreiber K. Sciascia S. de Groot P. G. Devreese K. Jacobsen S. Ruiz-Irastorza G. (2018). Antiphospholipid syndrome. *Nat. Rev. Dis. Primers* 4:18005. 10.1038/nrdp.2018.5 29368699

[B78] Schwartz M. Deczkowska A. (2016). Neurological disease as a failure of brain-immune crosstalk: the multiple faces of neuroinflammation. *Trends Immunol.* 37 668–679. 10.1016/j.it.2016.08.001 27616557

[B79] Shimizu H. Matsuguchi T. Fukuda Y. Nakano I. Hayakawa T. Takeuchi O. (2002). Toll-like receptor 2 contributes to liver injury by *Salmonella* infection through Fas ligand expression on NKT cells in mice. *Gastroenterology* 123 1265–1277. 10.1053/gast.2002.36006 12360487

[B80] Singh A. K. Wilson M. T. Hong S. Olivares-Villagomez D. Du C. Stanic A. K. (2001). Natural killer T cell activation protects mice against experimental autoimmune encephalomyelitis. *J. Exp. Med.* 194 1801–1811. 10.1084/jem.194.12.1801 11748281 PMC2193577

[B81] Skaper S. D. Facci L. Zusso M. Giusti P. (2018). An inflammation-centric view of neurological disease: beyond the neuron. *Front. Cell Neurosci.* 12:72. 10.3389/fncel.2018.00072 29618972 PMC5871676

[B82] Stax A. M. Tuengel J. Girardi E. Kitano N. Allan L. L. Liu V. (2017). Autoreactivity to sulfatide by human invariant NKT Cells. *J. Immunol.* 199 97–106. 10.4049/jimmunol.1601976 28526683

[B83] Sun B. L. Wang L. H. Yang T. Sun J. Y. Mao L. L. Yang M. F. (2018). Lymphatic drainage system of the brain: a novel target for intervention of neurological diseases. *Prog. Neurobiol.* 16 118–143. 10.1016/j.pneurobio.2017.08.007 28903061

[B84] Suzuki Y. Onodera H. Tago H. Saito R. Ohuchi M. Shimizu M. (2005). Altered populations of natural killer cell and natural killer T cell subclasses in myasthenia gravis. *J. Neuroimmunol.* 167 186–189. 10.1016/j.jneuroim.2005.06.015 16040133

[B85] Taniguchi M. Seino K. Nakayama T. (2003). The NKT cell system: bridging innate and acquired immunity. *Nat. Immunol.* 4 1164–1165. 10.1038/ni1203-1164 14639465

[B86] Teige A. Teige I. Lavasani S. Bockermann R. Mondoc E. Holmdahl R. (2004). CD1-dependent regulation of chronic central nervous system inflammation in experimental autoimmune encephalomyelitis. *J. Immunol.* 172 186–194. 10.4049/jimmunol.172.1.186 14688325

[B87] Teo W. Y. Elghetany M. T. Shen J. Man T. K. Li X. Chintagumpala M. (2014). Therapeutic implications of CD1d expression and tumor-infiltrating macrophages in pediatric medulloblastomas. *J. Neurooncol.* 120 293–301. 10.1007/s11060-014-1572-5 25115738

[B88] Thiruppathi M. Rowin J. Li Jiang Q. Sheng J. R. Prabhakar B. S. Meriggioli M. N. (2012). Functional defect in regulatory T cells in myasthenia gravis. *Ann. N.Y. Acad. Sci.* 1274 68–76. 10.1111/j.1749-6632.2012.06840.x 23252899 PMC3531815

[B89] van der Vliet H. J. von Blomberg B. M. Nishi N. Reijm M. Voskuyl A. E. van Bodegraven A. A. (2001). Circulating V(alpha24+) Vbeta11+ NKT cell numbers are decreased in a wide variety of diseases that are characterized by autoreactive tissue damage. *Clin. Immunol.* 100 144–148. 10.1006/clim.2001.5060 11465942

[B90] Van Kaer L. Wu L. Parekh V. V. (2015). Natural killer T cells in multiple sclerosis and its animal model, experimental autoimmune encephalomyelitis. *Immunology* 146 1–10. 10.1111/imm.12485 26032048 PMC4552496

[B91] Wang Y. H. Jia J. C. Liu G. Wang Y. F. (2015). Research on the influence of alpha-GalCer activating experimental autoimmune myasthenia gravis mice NKT cells at different times on myasthenia gravis. *J. Biol. Regul. Homeost. Agents* 29 195–200. 25864758

[B92] Wang Z. K. Xue L. Wang T. Wang X. J. Su Z. Q. (2016). Infiltration of invariant natural killer T cells occur and accelerate brain infarction in permanent ischemic stroke in mice. *Neurosci. Lett.* 633 62–68. 10.1016/j.neulet.2016.09.010 27637387

[B93] Wermeling F. Lind S. M. Jordo E. D. Cardell S. L. Karlsson M. C. (2010). Invariant NKT cells limit activation of autoreactive CD1d-positive B cells. *J. Exp. Med.* 207 943–952. 10.1084/jem.20091314 20439539 PMC2867286

[B94] Wong C. H. Jenne C. N. Lee W. Y. Leger C. Kubes P. (2011). Functional innervation of hepatic iNKT cells is immunosuppressive following stroke. *Science* 334 101–105. 10.1126/science.1210301 21921158

[B95] Wong C. H. Jenne C. N. Tam P. P. Leger C. Venegas A. Ryckborst K. (2017). Prolonged activation of invariant natural killer T cells and TH2-skewed immunity in stroke patients. *Front. Neurol.* 8:6. 10.3389/fneur.2017.00006 28154551 PMC5244395

[B96] Wu L. Van Kaer L. (2009). Natural killer T cells and autoimmune disease. *Curr. Mol. Med.* 9 4–14. 10.2174/156652409787314534 19199937

